# Low B and T lymphocyte attenuator expression on CD4^+^ T cells in the early stage of sepsis is associated with the severity and mortality of septic patients: a prospective cohort study

**DOI:** 10.1186/s13054-015-1024-4

**Published:** 2015-08-28

**Authors:** Rui Shao, Chun-Sheng Li, Yingying Fang, Lianxing Zhao, Chenchen Hang

**Affiliations:** Department of Emergency Medicine, Beijing Chao-yang Hospital, Capitcal Medical University, 8# Worker’s Stadium South Road, Chao-yang District, Beijing, 100020 China

## Abstract

**Introduction:**

B and T lymphocyte attenuator (BTLA) is an inhibitory receptor, whose primary role in CD4^+^ T cell is thought to inhibit cytokine production. We explore BTLA expression on CD4^+^ T cells in healthy controls and septic patients, and assess the correlation of BTLA expression on CD4^+^ T cells in the early stage of sepsis with the severity and mortality of septic patients in the emergency department (ED).

**Methods:**

336 consecutive patients were included in this study. BTLA expression on CD4^+^ T cells was measured by flow cytometry within 24h of ED admission.

**Results:**

Our results showed that the percentage of BTLA^+^/CD4^+^ T cells was high expression in healthy volunteers and it was statistically reduced in severe sepsis and septic shock compared with healthy controls(all P<0.01). The area under the receiver operating characteristic (AUC) curves of BTLA expression on CD4^+^ T cells was slightly lower than that of procalcitonin (PCT) and Mortality in Emergency Department Sepsis (MEDS) score. The percentage of BTLA^+^/CD4^+^T cells was lower in non-survivors than in survivors (P<0.01), and similar results were obtained when expressed as mean of fluorescence intensities (MFI) (P<0.01). Adjusted logistic regression analysis suggested that the percentage of BTLA^+^/CD4^+^ T cells was associated with 28-day mortality in septic patients (odds ratio (OR) = 0.394).

**Conclusion:**

Our study shows that the percentage of BTLA^+^/CD4^+^ T cells was high in healthy volunteers. Furthermore, lower percentage of BTLA^+^/CD4^+^ T cells during the early stage of sepsis is associated with the severity and the mortality of septic patients.

## Introduction

Sepsis, a systemic deleterious host inflammatory response to infection, has a high mortality rate, especially in patients with severe sepsis or septic shock [1]. Septic syndromes involve overstimulated host response and ineffective bacterial clearance [2, 3]. Immune system dysfunction is widely accepted as the main pathophysiological process in septic patients presenting as severely immunocomprised and inefficient at clearing invasive microbial pathogens [4, 5]. Negative co-inhibitory molecules, including programmed death receptor-1 (PD-1), B and T lymphocyte attenuator (BTLA) and other inhibitory molecules, play a major role in septic patients with immunosuppression [6].

BTLA is a recently characterized co-inhibitory molecule expressed on T cells, B cells, natural killer (NK) cells, macrophages and dendritic cells [7, 8]. It plays a role in regulating T cell function and attenuating pro-survival signaling in CD4^+^ T cells [8, 9]. Studies have shown that the level of expression of BTLA on circulating T lymphocytes is associated with nosocomial infections and mortality in sepsis [6, 10]. However, there are conflicting reports on the level of BTLA expression on CD4+ T cells in healthy controls and patients with sepsis [6, 10]. Additionally, the relationship between the level of BTLA expression on circulating CD4^+^ T lymphocytes and the severity of sepsis has not been elucidated. Furthermore, there have been no studies exploring whether BTLA expression on circulating CD4^+^ T lymphocytes is associated with the mortality of patients with sepsis. Given the above considerations, this prospective cohort study was designed to explore the level of BTLA expression on circulating CD4^+^ T lymphocytes in healthy volunteers and patients with sepsis. We also examined the correlation between the level of BTLA expression on circulating CD4^+^ T lymphocytes and the disease severity and mortality of patients with sepsis.

## Methods

### Patients

Data were collected between May 2014 and January 2015 from patients admitted to the Emergency Department (ED) of Beijing-Chao Hospital, an urban university tertiary hospital with about 250,000 ED admissions annually. Patients who were admitted to the ED on days 1 to 2 of the onset of the signs of systemic inflammatory response syndrome (SIRS) were evaluated for possible enrollment according to the inclusion and exclusion criteria. Eligible patients were categorized into groups according to the severity of disease (which included SIRS, sepsis, severe sepsis, and septic shock), and blood samples were obtained within 24 h of enrollment.

SIRS, sepsis, severe sepsis, and septic shock were diagnosed according to the diagnostic criteria of the 2001 SCCM/ESICM/ACCP/ATS/SIS International sepsis definitions conference [11]. SIRS was defined with at least two of the following criteria: (a) body temperature >38 °C or <36 °C, (b) heart rate >90 beats per minute, (c) respiratory rate >20 breaths per minute or arterial partial pressure of carbon dioxide (PaCO_2_) <32 mmHg, (d) white cell count >12,000/mm^3^ or <4,000/mm^3^, or the presence of >10 % immature neutrophils. Sepsis was defined by the presence of both infection and SIRS. Severe sepsis was defined as sepsis-induced hypotension or dysfunction. Septic shock was defined as sepsis-induced hypotension persisting despite adequate fluid resuscitation, and requiring vasopressor therapy. According to the criteria of the International Sepsis Forum Consensus Conference on Definitions of Infection [12], the infection was defined on the basis of clinical features, laboratory findings, and imaging tests. The criteria for organ dysfunction were as follows: sepsis-induced hypotension; lactate above normal upper limits; urine output <0.5 ml/kg/h for more than 2 h despite adequate fluid resuscitation, or creatinine >2.0 mg/dl (176.8 μmol/L); acute lung injury with PaO_2_/inspired oxygen fraction (FiO_2_) <250 mmHg in the absence of pneumonia as infection source, or acute lung injury with PaO_2_/FiO_2_ <200 mmHg in the presence of pneumonia as the infection source; bilirubin >2.0 mg/dl (34.2 μmol/L); platelet count <100,000/mm^3^, or international normalized ratio >1.5. The exclusion criteria were: (1) patient’s age <18 years; (2) patients with HIV infection, viral hepatitis, and other pre-existing hematological or immunological disease; (3) patients who declined to consent, and (4) patients with signs of SIRS occurring >3 days prior to admission, or patients who were transferred from another hospital.

Clinical characteristics of patients, including demographic characteristics (age and gender); vital signs (heart rate, respiratory rate, body temperature, and blood pressure), and past medical history and correlative laboratory examinations (white blood cell count (WBC), blood gas analysis, blood biochemistry, imaging examination and others) were collected upon enrollment. The mortality in emergency department sepsis (MEDS) score was calculated using the related clinical and demographic data [13]. Fifty age-matched healthy controls with no history or clinical disease were enrolled. This study was approved by the Beijing Chao-yang Hospital Ethics Committee. Written informed consents were obtained from the patients and volunteers. Consents for patients who were unable to consent were provided by first-degree relatives. According to 28-day survival, patients with sepsis, severe sepsis, and septic shock were classified as surviving or non-surviving. During the follow-up period, the enrolled patients who died from any cause were classified as non-survivors.

### Flow cytometry

**Fig. 1:**
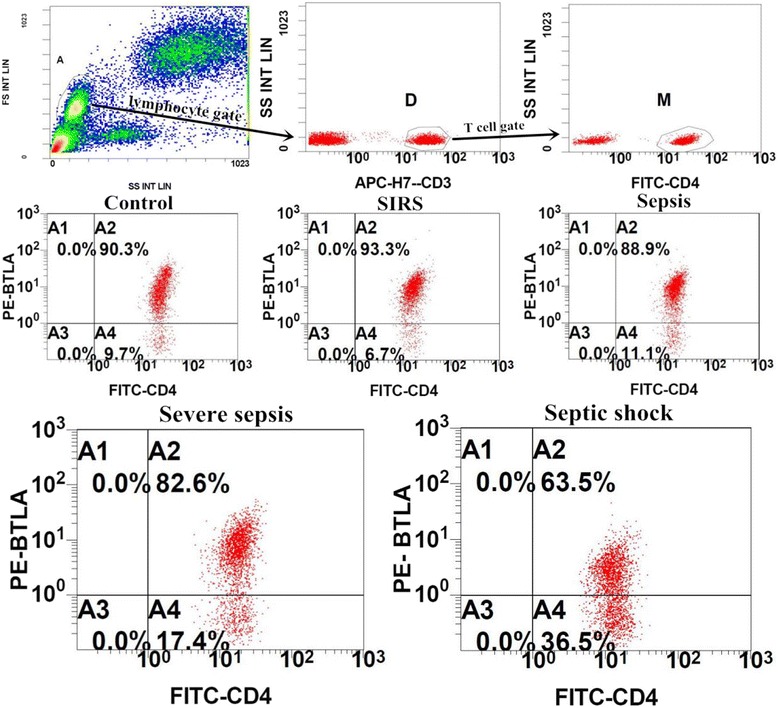
Representative flow dot plots of lymphocyte gating strategy and the percentage of B and T lymphocyte attenuator (*BTLA*)^+^/CD4^+^ T cells in five groups. All lymphocytes (*gate A*) were gated by forward scatter/side scatter (FSC/SSC), and then differentiating T cell subsets (gate *D*) based on CD3^+^ staining in lymphocytes, and then CD4^+^ T cells (*gate M*) were further identified by CD4^+^ staining in T cells . The multiple dot plots of CD4 vs BTLA are representative of the percentage of BTLA^+^/CD4^+^T cells in five groups (control, systemic inflammatory response syndrome (*SIRS*), sepsis, severe sepsis, and septic shock). *PE* phycoerythrin, *FITC* fluorescein isothiocyanate, *FSC INT LIN* forward scatter integral linear, *SS INT LIN* side scatter integral linear

Samples of peripheral blood were collected in ethylenediamine tetraaccetic acid (EDTA) anticoagulant tubes and transported to the laboratory at 4 °C within 1 h. The erythrocytes were lysed and cells were stained by researcher who was blinded to the clinical data. According to the manufacturer’s recommendations, monoclonal antibodies and their isotype controls were used: phycoerythrin (PE)-labeled anti-BTLA (5 μl, clone J168-540; BD Bioscience, San Jose, CA, USA), APC-H7 labeled anti-CD3 (5 μl, clone SK7; BD Bioscience), fluorescein isothiocyanate (FITC)-labeled anti-CD4 (clone OKT4; eBioscience, San Diego, CA, USA) per 100 μl of whole blood. Samples were run on the Gallios™ Flow Cytometer (Beckman Coulter, Inc.) and analyzed using Gallios Software Version 1.0 (Beckman Coulter, Inc.). Lymphocytes were gated by forward scatter (FSC) and side scatter (SSC), and T cells subsets were further identified by CD3^+^ and CD4^+^ staining (Fig. 1). At least 5,000 CD4^+^ T lymphocytes were analyzed from each sample. Threshold was defined with the isotype control. Results are expressed as percentage of BTLA^+^/CD4^+^ T cells and its mean of fluorescence intensities (MFI).

### Statistical analysis

The baseline characteristics are described as frequencies, percentages, medians and inter-quartile ranges. Kruskal-Wallis one-way analyses of variance was performed for multi-group comparisons. The groups were compared using the Pearson chi squared (χ^2^) test for categorical data and the Dunnett test (a post-hoc multiple comparisons test) for continuous variables. Receiver operating characteristic (ROC) curves were established and the area under the ROC curve (AUC) was determined to evaluate the predictive qualities of BTLA expression on CD4^+^ T cells. Differences in the AUC values were analyzed using the *Z* test formula. Binary logistic regression was used to analyze the variables associated with 28-day mortality. All statistical tests were two-tailed, and *P* values of 0.05 or less were considered statistically significant. All data were analyzed using SPSS 19.0 software (SPSS Inc., Chicago, IL, USA).

## Results

### Characteristics of enrolled subjects

In total, 286 patients who met the eligibility criteria and 50 healthy controls were enrolled. There were no significant differences in age, gender, or correlative diseases among the patients in the five groups (control, SIRS, sepsis, severe sepsis, and septic shock groups) (Table 1). The baseline characteristics, diseases and associated infections of all participants are also shown in Table 1. The median values of WBC, absolute lymphocyte count (ALC), procalcitonin (PCT), and MEDS score in each group were significantly different. Notably, our results showed that ALC was obviously decreased in patients with sepsis. A summary of the values is shown in Table 1.

**Table 1:** Baseline characteristics of the study population

	Control	SIRS	Sepsis	Severe sepsis	Septic shock	*P* value
Number	50	59	93	71	63	
Age, years	68 (66−74)	70 (59−78)	71 (66−78)	73 (62−77)	74 (64−79)	0.34
Male, %	62.0	57.6	60.2	63.4	60.3	0.97
WBC, ×10^9^/L	6.6 (5.6−7.6)	9.2 (8.1−11.6)	11.9 (9.6−14.2)	14.9 (10.9−17.7)	16.6 (13.5−19.6)	<0.001
Lymphocytes, ×10^9^/L	2.8 (2.4−3.1)	1.4 (1.0−1.7)	1.1 (0.9−1.7)	0.8(0.5−1.2)	0.6 (0.4−0.9)	<0.001
PCT, g/ml	0.06 (0.05−0.1)	0.2 (0.05−0.3)	1.0 (0.2−2.1)	4.6 (0.9−8.1)	8.3 (1.8−22.3)	<0.001
MEDS score	0	5 (3−7)	9 (6.5−11)	12 (9−16)	19 (16−23)	<0.001
Percentage of BTLA^+^/CD4^+^T cells						
	90.3 (85.6−91.9)	91.3 (89.0−93.2)	88.4 (81.9−91.3)	86.5 (78.6−90.1)	79.5 (63.6−86.6)	<0.001
MFI of BTLA on CD4^+^ T cells						
	7.2 (6.0−8.1)	10.2 (8.7−13.3)	8.7 (7.0−11.0)	7.6 (6.2−8.9)	6.5 (4.9−8.7)	<0.001
Diagnosis (number)						
Respiratory		AECOPD (27)	Pneumonia (58)	Pneumonia (58)	Pneumonia (58)	0.80
		Asthma (14)				
Abdominal		Pancreatitis (8)	IAI (17)	IAI (13)	IAI (11)	0.88
Urinary			Pyelonephritis (4)	Pyelonephritis (7)	Pyelonephritis (4)	0.36
Cerebral		Stroke (4)	Meningitis (9)	Meningitis (5)	Meningitis (9)	0.44
Others		DKA (6)	Skin/soft tissue infection (5)			
28-day mortality, number (%)		5 (8.5 %)	18 (19.5 %)	33 (46.5 %)	37 (58.7 %)	<0.001

Data are shown as median and interquartile range (IQR) unless stated otherwise. *WBC* white blood cells, *PCT* procalcitonin, *MEDS* Mortality in Emergency Department Sepsis, *BTLA* B and T lymphocyte attenuator, *MFI* mean of fluorescence intensities, *AECOPD* acute exacerbation of chronic obstructive pulmonary disease, *IAI* intraabdominal infection, *DKA* diabetic ketoacidosis, *ED* Emergency Department, *SIRS* systemic inflammatory response syndrome

### BTLA expression on CD4^+^T lymphocytes

**Fig. 2:**
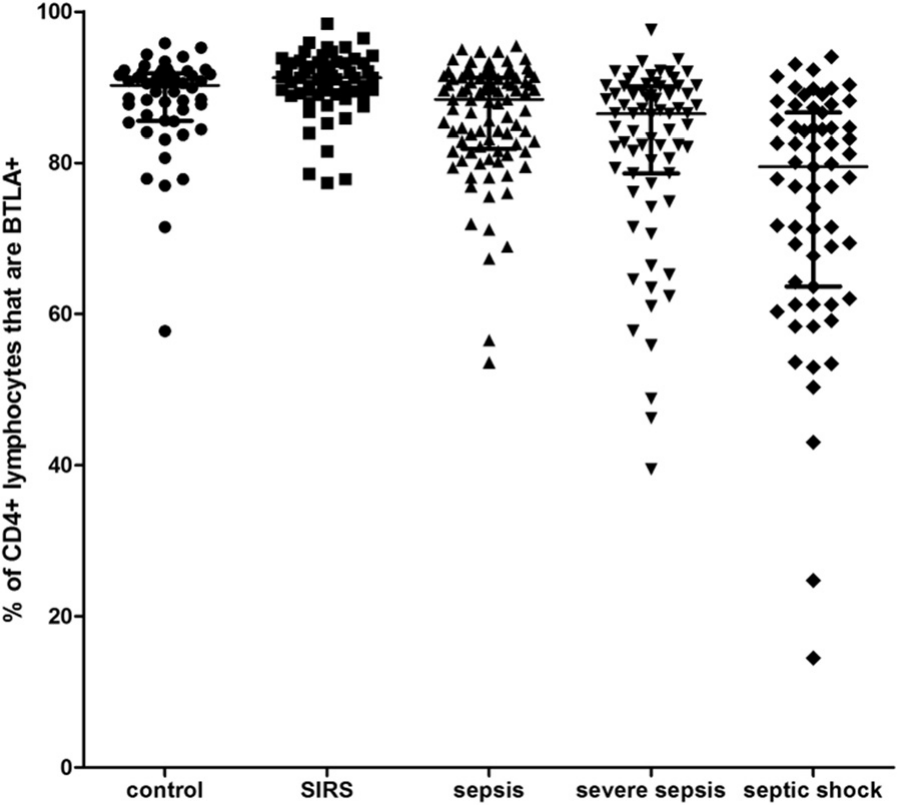
The percentage of B and T lymphocyte attenuator (*BTLA*)^+^/CD4^+^ T cells in five groups. There was a stepwise reduction in the percentage of BTLA^+^/CD4^+^T cells from patients with systemic inflammatory response syndrome (*SIRS*) compared to patients with sepsis, severe sepsis, or septic shock

The median levels of BTLA expression on CD4^+^T cells in each group are presented in Table 1. The median value of the percentage of BTLA^+^/CD4^+^T cells in healthy volunteers was 90.2 % (85.5−91.9 %). Interestingly, we observed that the percentage of BTLA^+^/CD4^+^T cells was significantly reduced in patients with severe sepsis or septic shock compared with healthy controls (all *P* <0.01). This was also obviously reduced in patients with septic shock compared with patients with severe sepsis (*P* <0.05), However, there were no differences in the percentage of BTLA^+^/CD4^+^T cells between patients with SIRS and healthy volunteers or between patients with sepsis and healthy controls (*P* >0.05). There was a stepwise reduction in the percentage of BTLA^+^/CD4^+^T cells within 24 h of ED admission in patients with SIRS compared with patients with sepsis, severe sepsis, or septic shock (*P* <0.01; Figs. 1 and 2). We observed that the MFI of BTLA on CD4^+^ T cells was significantly higher in patients with SIRS or sepsis than in healthy controls (*P* <0.01), but there was no statistically significant difference between the MFI of BTLA on CD4^+^ T cells in patients with severe sepsis or septic shock and healthy controls (all *P* >0.05).

**Fig. 3:**
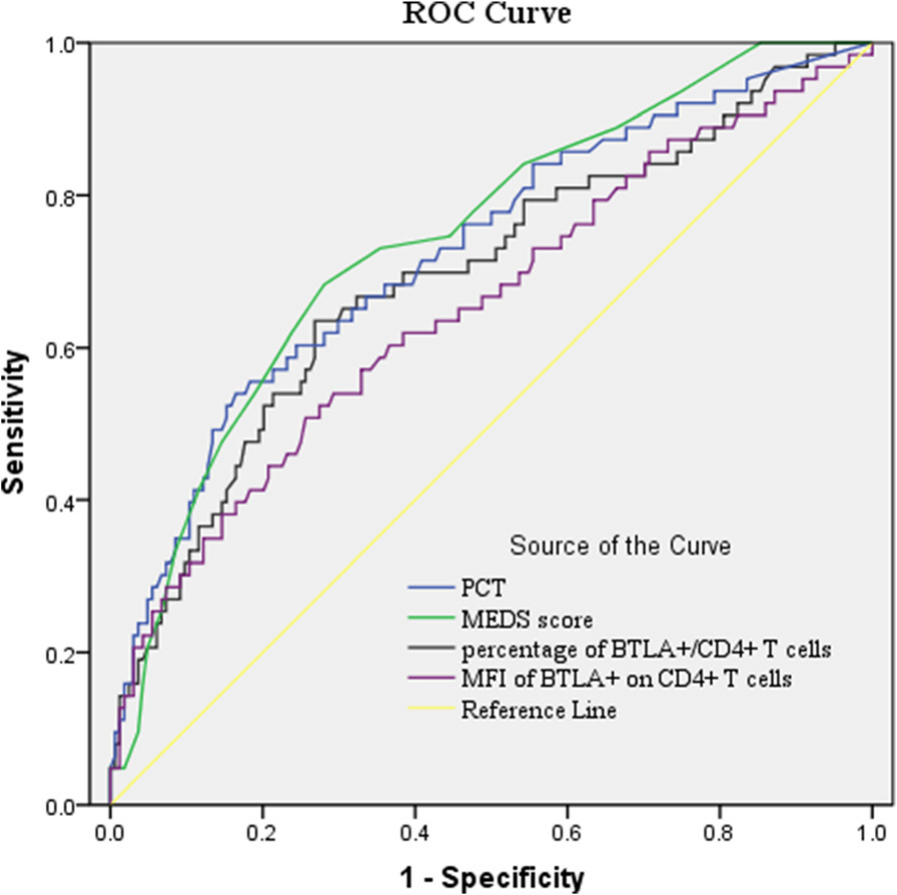
Receiver operating characteristic (ROC) curve for predicting 28-day mortality in patients with sepsis. Areas under the curve (AUCs) for predicting 28-day mortality: procalcitonin (*PCT*) (*blue line*) 0.726 (95 % CI: 0.647 to 0.785), mortality in emergency department sepsis (*MEDS*) score (*green line*) 0.743 (95 % CI: 0.672 to 0.814), percentage of B and T lymphocyte attenuator (*BTLA*)+/CD4+ T cells (*black line*) 0.693 (95 % CI: 0.613 to 0.773), mean of fluorescence intensities (*MFI*) of BTLA on CD4+ T cells (*purple line*) 0.653 (95 % CI: 0.569 to 0.736)

The AUC of the percentage of BTLA^+^/CD4^+^ T cells for predicting 28-day mortality was 0.693, which was not statistically significantly different compared with the MEDS score (0.743) or PCT (0.726) according to the *Z* test (all *P* >0.05; Fig. 3). The ROC curve analysis showed that the optimal threshold for predicting 28-day mortality in patients was 82.25 % of BTLA^+^/CD4^+^ T cells (sensitivity 53.4 %, specificity 72.7 %, positive predictive value (PPV) 55.3 %, negative predictive value (NPV) 71.1 %). Similarly, the AUC of the MFI of BTLA expression on CD4+ T cells for predicting 28-day mortality was 0.653, which was slightly lower than that of the MEDS score (0.743) or PCT (0.726) according to the *Z* test (all *P* >0.05; Fig. 3). The best cutoff of the MFI of BTLA expression on CD4+ T cells for predicting 28-day mortality in patients was 7.54 (sensitivity 62.5 %, specificity 64 %, PPV 52.4 %, NPV 73 %).

**Fig. 4:**
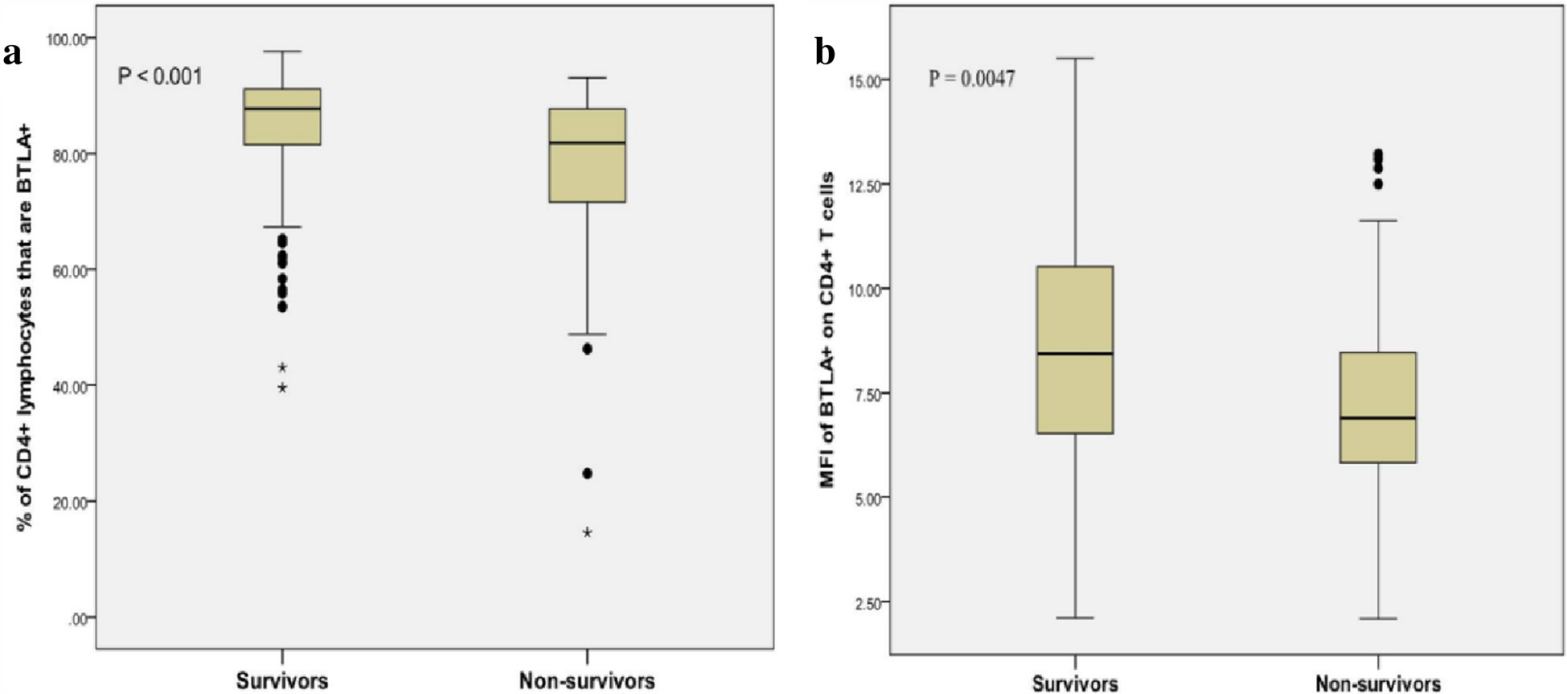
Box-plot representation of the percentage of B and T lymphocyte attenuator (BTLA)+/CD4+ T cells (**A**) and mean of fluorescence intensities (MFI) of BTLA+ on CD4+ T cells (**B**) according to outcome. Data are shown as box plot with medians (*lines inside boxes*), 25th and 75th quartiles (*limits of boxes*) and *whiskers* indicate the range. The median of the percentage of BTLA^+^/CD4^+^ T cells or its MFI were higher in survivors (n = 139) than in non-survivors (n = 88)

The percentage of BTLA^+^/CD4^+^T cells was lower in non-survivors compared with survivors (*P* <0.01). Similar results were obtained when expressed as MFI of BTLA on CD4^+^ T cells (*P* <0.01; Fig. 4).

In multivariate analysis, the logistic regression model was adjusted for age, gender, WBC, ALC, PCT concentrations, the MEDS score, the percentage of BTLA^+^/CD4^+^T cells , and MFI of BTLA on CD4^+^ T cells, to determine the relationship of 28-day mortality in patients with sepsis, severe sepsis and septic shock. The model was determined by the method of ENTER (default with the menu system). After univariate analysis, the MEDS score, PCT, ALC, the percentage of BTLA^+^/CD4^+^T cells, and MFI of BTLA on CD4+ T cells were included in the final model (*P* <0.05). Using multivariate logistic regression analysis, the percentage of BTLA^+^/CD4^+^T cells (odds ratio (OR) = 0.869, 95 % CI: 0.769 to 0.981, *P* = 0.023) is associated with the 28-day mortality, but MFI of BTLA on CD4^+^ T cells was not (OR = 0.900, 95 % CI: 0.789 to 1.025, *P* = 0.113). The detailed results are illustrated in Table 2.

**Table 2:** Independent factors predicting 28-day mortality in patients with sepsis, severe sepsis, and septic shock

Variable	B	SE	Wald	*P* value	Odds ratio	95 % CI for EXP (B)	
						Lower limit	Lower limit
Percentage of BTLA^+^/CD4^+^ T cells	−0.141	0.062	5.145	0.023	0.869	0.769	0.981
MFI of BTLA on CD4^+^ T cells	−0.106	0.067	2.508	0.113	0.900	0.789	1.025
MEDS	0.180	0.033	29.358	<0.001	1.197	1.121	1.277
PCT	0.022	0.015	2.206	0.137	1.103	0.993	1.054
ALC	0.256	0.277	0.856	0.355	1.292	0.751	2.221
Constant	−2.155	0.756	8.120	0.004	0.116		

*BTLA* B and T lymphocyte attenuator, *MFI* mean of fluorescence intensities, *MEDS* mortality in emergency department sepsis score, *PCT* procalcitonin, *ALC* absolute lymphocyte count

## Discussion

The major findings of this study are the unexpected results. We showed that the percentage of BTLA^+^/CD4^+^ T cells was high in healthy volunteers. In the early stage of sepsis, there was a stepwise reduction in the percentage of BTLA^+^/CD4^+^T cells in patients with SIRS compared with patients with sepsis, severe sepsis, or septic shock. The percentages of BTLA^+^/CD4^+^T cells were significantly reduced in patients with severe sepsis or septic shock compared with healthy controls. Therefore, in the early stage of sepsis, the percentage of BTLA^+^/CD4^+^T cells may contribute to the risk stratification for sepsis. Additionally, the percentage of BTLA^+^/CD4^+^T cells was lower in non-survivors than in survivors, and it was associated with 28-day mortality in patients with sepsis. Our findings suggest that not only did the percentage of BTLA^+^/CD4^+^T cells correlate with the severity of sepsis, but also it was valuable for prognostic evaluation of sepsis. Therefore, the percentage of BTLA^+^/CD4^+^T cells was a predictor of pejorative outcome in patients with sepsis as well. The unexpected results were inconsistent with previous studies.

Sepsis is a complicated immune response, and in general it involves massive release of inflammatory mediators during the early stage, followed by rapid development of an immunosuppression state [4, 14]. Immunosuppression that develops late in the disease course of septic syndromes presents as inability to clear the initial infection and a loss of the delayed hypersensitivity response [14, 15]. Co-inhibitory molecules act as negative regulators of TCR-mediated activation and cytokine releases during immune responses. In the early stage of sepsis, expression of inhibitory molecules is increased to prevent the cytokine storm from inducing organ failures. However, during the immunosuppression stage, high expression of inhibitory molecules may be detrimental through the subsequent induction of T cell anergy or immune cell deactivation. BTLA was initially described as a co-inhibitory receptor, whose primary roles in CD4^+^ T cells were thought to be the inhibition of cytokine production and maintenance of a state of immune tolerance to self-antigens [16, 17]. However, Hurchla and colleagues found a pro-survival role of BTLA in an in vivo model of chronic allostimulation [18]. A study by Shubin and colleagues showed that increased BTLA^+^CD4^+^ lymphocytes were found in patients who developed a subsequent infection, and that BTLA may be a potential biomarker and mediator of sepsis-induced immunosuppression [10]. The differences between our study and that of Shubin and colleagues are detailed below.

Our results showed that the percentage of BTLA^+^/CD4^+^T cells in healthy volunteers was about 90 %, while Shubin and colleagues found the percentage of BTLA^+^/CD4^+^T cells in healthy controls to be about 50 %. The discrepancy between our results and those of Shubin and colleagues may be due to the fact that their sample size of healthy controls (n = 6) was relatively small. Conversely, the percentage of BTLA^+^/CD4^+^T cells in healthy controls from the study by Zhang and colleagues was in agreement with ours [19]. There is another hypothesis. BTLA gene polymorphisms were not present at the same frequencies in the Chinese Han and the Caucasian population, and the basal expression of BTLA on CD4^+^ T cell may be different between various lineages. For example, several minor alleles of BTLA in the Caucasian lineage are major alleles in the Han Beijing population [20]. Shubin and colleagues also measured the percentage of BTLA^+^/CD4^+^T cells in patients with SIRS or sepsis. Interestingly, they found that patients with sepsis had a significantly increased percentage of BLTA^+^CD4^+^ lymphocytes compared with patients with SIRS. The discrepancies between our findings and those of shubin and colleagues may be due to the timing of the sample acquisition. The samples from Shubin and colleagues were collected at different times after ICU admission and the patients had already been subjected to standardized therapy in the ICU. The median number of sampling times for patients with sepsis were significantly longer than those of patients with SIRS (21 vs 4), whereas our patient samples were collected within 24 h of ED admission. Therefore, the patients in the Shubin study may already have been in the immunosuppression stage of sepsis, whereas our patients were still in the early stages of sepsis. Additionally, whether the levels of BTLA expression on CD4^+^ T cells change over time remains unknown. However, one study showed that the level of PD-1 expression on CD8^+^ T cells increased over time during sepsis [21]. Therefore, it is possible that the level of BTLA expression also varies and further studies are needed to certify this.

Unlike other co-inhibitory receptors, our results suggested that lower levels of BTLA expression on CD4^+^ T cells during the early stage of sepsis may increase the risk of pejorative outcome. Many studies on BTLA have indicated similar results. Treatment with an agonistic anti-BTLA antibody was found by Albring and colleagues to prevent graft-versus-host disease [22]. Oya and colleagues found that BTLA inhibited lipopolysaccharide (LPS)-induced endotoxic shock and that agonistic anti-BTLA antibody had therapeutic potential for endotoxic shock [23]. Collectively, these studies suggested that high levels of BTLA expression may play a protective role during the early stage of sepsis.

Although both proinflammatory and anti-inflammatory processes begin promptly after the initiation of sepsis, in general there is predominance of an initial hyperinflammatory phase [24]. The expression of inhibitory molecule was upregulated to prevent the cytokine storm in the early stage of the host response from inducing organ failure. However, it was detrimental if the expression of inhibitory molecule persisted during the stage of immunosuppression. The primary role of BTLA in CD4^+^ T cells is thought to be suppressing proflammatory responses [17]. Therefore, low BTLA expression during the first stage of sepsis may result in an over-zealous proinflammatory response which might be as detrimental as high expression at latter stages. The study by Oya and colleagues found that BTLA suppresses cytokine production in innate immune cells and that BTLA-deficient mice are highly susceptible to LPS-induced shock [23]. In contrast to other inhibitory molecules, the changes in BTLA expression during the early stage of sepsis may be more useful for evaluating the prognosis of patients with sepsis. However, scarce data are available on BTLA expression in patients with sepsis and further studies are required for understanding the molecular mechanisms.

In this study, BTLA expression on CD4+ T cells was a reliable outcome predictor, similar to the MEDS score and better than PCT. Of note, our results showed that ALC was obviously decreased in patients with sepsis, which is consistent with the study by Castelino and colleagues, who reported that lymphopenia (often at levels of less than half of the reference value) in hospitalized patients is most frequently caused by acute illness, notably sepsis and trauma [25]. Similarly, Jahangiri and colleagues reported that patients with sepsis had a significant decrease in their ALC (53 % lower compared with controls) [26]. It remains unclear if lymphocyte apoptosis occurs at the onset of sepsis, so this observation should be explored in future studies.

It is important to distinguish between the functions of BTLA in the innate and adaptive immune response. The innate immune response is the host’s first line of defense against infection and it is essential for the initiation of adaptive immune response. Sun and colleagues showed that BTLA-deficient mice exhibited significantly higher bacterial clearance in the early innate immunity [27]. Shubin and colleagues demonstrated that BTLA expression contributed to septic morbidity and mortality in the early phase of immune response [28]. However, it has also been found that BTLA inhibits LPS-induced endotoxic shock and proinflammatory cytokine production [21]. BTLA also plays an important role in acquired immune responses. Oya and colleagues showed that BTLA-deficiency mice are susceptible to developing autoimmune hepatitis-like disease [29]. Administration of agonistic anti-BTLA antibody has been shown to prevent graft-versus-host disease [22]. Our results showed that low levels of BTLA expression may be a risk factor for septic mortality. Additional studies examining the BTLA expression during both the innate and adaptive immunity will be required to fully understand the roles of BTLA in patients with sepsis.

Our study has several limitations. First, though it had a relatively large sample size, it was a single-center study and the findings need to be confirmed by a multicenter study. Second, the dynamic changes in the levels of BTLA expression on CD4^+^ T cells and the absolute number for BTLA expression on CD4^+^ T cells remain targets for future study. Third, our multivariate analysis has been adjusted for several potential confounding factors, but some risk factors, such as nutritional status, could not be considered. Poor nutritional status may be a major factor in the aggravation of sepsis, and a previous study has shown that malnutrition has several effects on host immunity [30]. Finally, this study was only an observational study and further studies are required to elaborate on the molecular mechanisms responsible for our findings.

## Conclusions

In conclusion, our study shows that the percentage of BTLA^+^/CD4^+^ T cells was high in healthy volunteers. Furthermore, a lower percentage of BTLA^+^/CD4^+^ T cells during the early stage of sepsis is associated with the severity of sepsis and the mortality of patients with sepsis.

## Key messages

- The percentage of BTLA^+^/CD4^+^ T cells was high in healthy volunteers
- A lower percentage of BTLA^+^/CD4^+^ T cells in the early stage of sepsis is associated with the severity of sepsis
- The percentage of BTLA^+^/CD4^+^T cells was lower in non-survivors than in survivors, and similar results were obtained when expressed as MFI
- The percentage of BTLA^+^/CD4^+^ T cells in the early stage of sepsis is associated with 28-day mortality in patients with sepsis, severe sepsis and septic shock

## Abbreviations

ALC: absolute lymphocyte count
AUC: area under the receiver operating characteristic curve
BTLA: B and T lymphocyte attenuator
CI: confidence interval
ED: Emergency Department
FiO_2_: inspired oxygen fraction
FITC: fluorescein isothiocyanate
FSC: forward scatter
LPS: lipolysaccharide
MEDS: mortality in emergency department sepsis
MFI: mean of fluorescence intensities
NPV: negative predictive value
OR: odds ratio
PaCO_2_: arterial partial pressure of carbon dioxide
PCT: procalcitotin
PD-1: programmed death receptor-1
PE: phycoerythrin
PPV: positive predictive value
ROC: receiver operating characteristic
SIRS: systemic inflammatory response syndrome
SSC: side scatter
WBC: white blood count
